# Enhanced setup for wired continuous long-term EEG monitoring in juvenile and adult rats: application for epilepsy and other disorders

**DOI:** 10.1186/s12868-019-0490-z

**Published:** 2019-03-04

**Authors:** Yasser Medlej, Rita Asdikian, Lara Wadi, Houssein Salah, Laura Dosh, Rabih Hashash, Nabil Karnib, Mohammad Medlej, Hala Darwish, Firas Kobeissy, Makram Obeid

**Affiliations:** 1 Department of Anatomy, Cell Biology and Physiological Sciences, Faculty of Medicine, American University of Beirut, Diana Tamari Sabbagh (DTS) Building, first floor, 117b, P.O. Box 11-0236, Riad El-Solh, Beirut, 1107 2020 Lebanon; 2 Faculty of Medicine, American University of Beirut, Beirut, Lebanon; 3 Animal Care Facility, American University of Beirut, Beirut, Lebanon; 4 Department of Natural Sciences, School of Arts and Sciences, Lebanese American University, Byblos, Lebanon; 5 Faculty of Fine Arts, Lebanese University, Beirut, Lebanon; 6 Rafic Hariri School of Nursing, American University of Beirut, Beirut, Lebanon; 7 Department of Biochemistry and Molecular Genetics, Faculty of Medicine, American University of Beirut, Beirut, Lebanon; 8 Division of Child Neurology, Department of Pediatric and Adolescent Medicine, American University of Beirut Medical Center, Beirut, Lebanon

**Keywords:** Electroencephalogram, Long-term, Continuous monitoring, Juvenile rats, Wired EEG, Swivel, Balance

## Abstract

**Background:**

The electroencephalogram (EEG) is a widely used laboratory technique in rodent models of epilepsy, traumatic brain injury (TBI), and other neurological diseases accompanied by seizures. Obtaining prolonged continuous EEG tracings over weeks to months is essential to adequately answer research questions related to the chronobiology of seizure emergence, and to the effect of potential novel treatment strategies. Current EEG recording methods include wired and the more recent but very costly wireless technologies. Wired continuous long-term EEG in rodents remains the mainstay approach but is often technically challenging due to the notorious frequent EEG cable disconnections from the rodent’s head, and to poor signal-to-noise ratio especially when simultaneously monitoring multiple animals. Premature EEG cable disconnections and cable movement-related artifacts result from the animal’s natural mobility, and subsequent tension on the EEG wires, as well as from potential vigorous and frequent seizures. These challenges are often accompanied by injuries to the scalp, and result in early terminations of costly experiments.

**Results:**

Here we describe an enhanced customized swivel-balance EEG-cage system that allows tension-free rat mobility. The cage setup markedly improves the safety and longevity of current existing wired continuous long-term EEG. Prevention of EEG cable detachments is further enhanced by a special attention to surgical electrode anchoring to the skull. In addition to mechanically preventing premature disconnections, the detailed stepwise approach to the electrical shielding, wiring and grounding required for artifact-free high signal-to-noise ratio recordings is also included. The successful application of our EEG cage system in various rat models of brain insults and epilepsy is described with illustrative high quality tracings of seizures and electrographic patterns obtained during continuous and simultaneous monitoring of multiple rats early and up to 3 months post-brain insult.

**Conclusion:**

Our simple-to-implement key modifications to the EEG cage setup allow the safe acquisition of substantial high quality wired EEG data without resorting to the still costly wireless technologies.

**Electronic supplementary material:**

The online version of this article (10.1186/s12868-019-0490-z) contains supplementary material, which is available to authorized users.

## Background

Electroencephalography (EEG) is a widely used technique and the main tool employed in the characterization of electrographic patterns and seizures for the diagnosis and treatment of acquired and genetic human epileptic disorders [1–3]. In the experimental arena, the EEG is also an essential tool used in preclinical research to study electrographic changes and seizures in rodent models that echo human diseases including hypoxic seizures, temporal lobe epilepsy and traumatic brain injury (TBI) [4–6]. The ability to record continuous EEGs with or without video monitoring in small animals, commonly in rodents, over weeks to months is essential to model human diseases, and to study the natural history of acquired or genetic epilepsies. This is particularly true for conditions such as TBI where the chronobiology of seizure emergence remains poorly understood [7, 8]. Investigating desperately needed novel anti-seizure and neuroprotective treatment strategies against acquired epilepsy, including post-traumatic epilepsy (PTE) is one area of translational research that is the focus of many laboratories including ours [6, 9, 10]. This specific area of research heavily relies on the ability to efficiently record continuous long-term EEG on multiple animals in order to assess the effect of novel interventions and drugs against the emergence of chronic spontaneous recurrent seizures; a process referred to as epileptogenesis [11]. More recently, long-term EEG data is also being increasingly subjected to fine computational analyses, mostly in an attempt to detect electrographic biomarkers that may predict seizure emergence [12].

Unfortunately, performing safe and prolonged EEG in rodents is complicated by a slew of challenges, the most notorious of which is disconnections of the EEG cable from the rodent’s head. Disconnections are due to the high mobility of the tethered rodents with resulting tension on the EEG cables, as well as to their natural tendency to chew on these cables [5]. This long-standing challenge to investigators not only compromises costly experiments, but studies may be terminated early and rodents may be injured, especially when they experience vigorous seizures. Some of the most widely used pre-clinical rodent models for studying chronic epilepsy after brain insults include hypoxia, TBI, chemoconvulsant administration, and electrical kindling [13–16]. These epileptogenic brain insults often lead to vigorous and prolonged seizures known as status epilepticus, which is followed by the emergence of spontaneous seizures after weeks to months (chronic epileptic phase). Vigorous convulsions associated with the initial status epilepticus or the subsequent seizures in the chronic phase put the animals at a high risk of injury secondary to the pulling tension that they exert on EEG cables. While high quality prolonged video-EEG recordings are even more needed when animals experience seizures, the risk of motion artifact and premature experimental termination is also the greatest during that time. Early termination of experiments results in loss of data, animal injuries, and inflicts losses on investigators’ time, energy, and budget. In addition to durability-related issues, another common technical challenge is the poor signal-to-noise ratio. Excessive artifact is usually due to environmental electrical noise, rodents’ high mobility, and difficulties in electrically shielding animals. This is especially true when simultaneously monitoring multiple rodents with the conventional medical EEG headboxes used in humans and; thus, designed to process, ground and reference EEG signal from only one individual subject.

Despite the recent emergence of wireless EEG technologies, “*wired*” EEG remains the most widely employed technique in rodents due to the usually limited number of implantable electrodes in wireless transmission technologies, as well as to the excessive cost of wireless transmitters [17]. This cost issue is compounded by the fact that large animal cohorts are needed in translational research, and consequently, a large number of expensive wireless transmitters is required. In this work, we describe an enhanced rodent EEG monitoring unit with a simple-to-build swivel-balance cage system that markedly improves the longevity of long-term standard “wired” EEG. We have designed a vertically pivoting swivel that accommodates rat movements in both the horizontal and vertical planes, and; thus, prevents premature disconnections and confers resistance against wear and tear that results from EEG cable torsion and tension. In addition to mechanically preserving electrical continuity and preventing premature disconnections, a special attention was dedicated to electrical shielding in our customized EEG cage system. Nonconductive material was used in building the cage and rack systems in order to efficiently electrically shield rats from each other, and from ambient electrical noise, which along with accurate electrode grounding and referencing, maximized signal-to-noise ratio and produced high-quality artifact-free continuous prolonged recordings simultaneously on multiple rats.

## Methods

### Animals and surgical implantation of epidural or depth EEG electrodes

Surgeries and all animal care and postoperative treatments were approved by, and conducted in compliance with the guidelines of the Institutional Animal Care and Use Committee (IACUC) at the American University of Beirut (AUB). The operations of IACUC at AUB are in compliance with the public health service policy on the humane care and use of laboratory animals (USA), and adopts the guidelines for the care and use of laboratory animals of the Institute for Laboratory Animal Research of the National Academy of Sciences (USA). Sprague–Dawley male rats were obtained from the Animal care facility at AUB. Prior to the experiments, rats were housed in high-temperature PolySulfone rectangular cages (42.5L × 27.6W × 153H cm) (TechniPlast, USA) up to 3–4 per cage, and then after electrode placement, they were individually placed in the customized Plexiglas single EEG cages described in the “Cages and racks” section. The rodent long-term EEG monitoring unit is a temperature-controlled room (23 °C) maintained on a 12 h light–dark cycle. Rats were fed in ad libitum (Teklad diet, Envigo, USA) and the cage bedding consisted of Combo 1/4″ & 1/8″ Bed-o’cobs blend (Andersons, USA). Depending on the experimental paradigm, electrode surgeries for EEG recordings are performed in our laboratory between P35 (postnatal day 35) and P65.

The following steps are followed in order to place epidural or depth electrodes.

1. The intramuscular surgical anesthesia mixture consists of ketamine (60 mg/kg), xylazine (6 mg/kg), and acepromazine (1.25 mg/kg).
2. After shaving the hair from the flat of the nose between the eyes down to the neck, the rat’s head is tightly secured on the stereotaxic frame (David Kopf Instruments, USA). Lubricating eye ointment is applied to prevent drying out and possible eye irritation from the used disinfectants.
3. The scalp is then sterilized with 10% iodine solution followed by 70% ethanol using cotton swabs through circular outward movements starting from the center of the shaved area. Using a sterile surgical blade, a 2 cm single midline incision is made starting from the bridge of the nose down to the posterior end of the cranium. The skin is held with a retractor, the calvarium is exposed, and bleeding controlled with cauterization and the application of pressure as needed. The exposed skull is then cleaned and dried using few drops of 3% hydrogen peroxide.
4. Once the skull surface is dry, 5 holes, 1.4 mm in diameter each, are made with a stereotaxic high-speed drill (David Kopf Instrument, USA) in order to place a combination of 5 epidural or depth electrodes in the skull (including one reference electrode). The screw size used as epidural electrode (outer diameter of 1.6 mm and length of 4.5 mm) is slightly larger than the drill bit (1.4 mm in diameter) to ensure tight secure screw anchoring to the skull. In our experimental paradigms, epidural electrodes are placed based on coordinates from the Sherwood and Timiras stereotaxic atlas of the developing rat brain, or the Watson and Paxinos atlas for adult rats. The screws or depth electrodes are attached to gold-plated stainless steel sockets (model E363/0, Plastics One, USA) via insulated 1.5–2 cm copper wires (0.3 mm in diameter). The 5 sockets are inserted in a 6-channel pedestal (MS363, Plastics One, USA), along with a 6th socket attached to a free 2 cm plastic-sheathed wire (0.5 mm in diameter) with an exposed 4 mm uninsulated tip that is looped and placed under the skin of the neck serving as a ground electrode. The pedestal and the screws are then covered with acrylic dental cement (Nic Tone^®^, MDC Dental, Mexico). Enough dental cement is poured to fill the entire incision site giving the “headset” its final shape without requiring any skin suturing (Fig. 1).

**Fig. 1:**
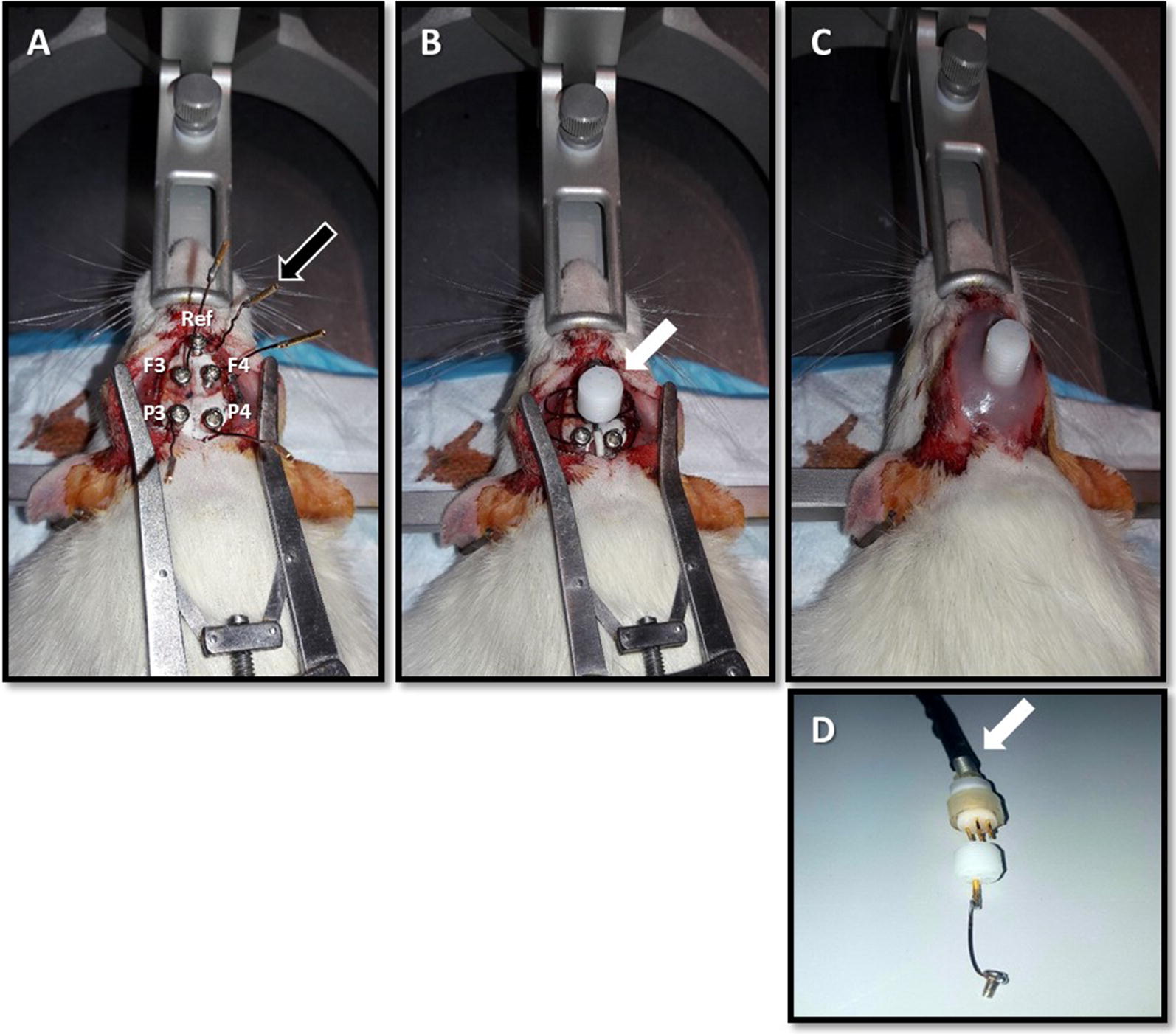
Surgical electrode implantation and headset assembly. Shown is an illustration of a set of epidural screw electrode placement in a P35–40 rat. **A** The five screw electrodes include two frontal (F3: left frontal, F4: right frontal, 2 mm anterior, and 3 mm lateral to the bregma), two parietal (P3: left parietal, P4: right parietal, 5 mm posterior, and 3 mm lateral to the bregma) and one anterior reference electrode (Ref, midline and 6 mm anterior to the bregma). The coordinates are based on the Sherwood and Timiras stereotaxic atlas of the developing brain. A sixth electrode consists of a free wire placed under the neck’s skin and serves as a ground electrode. Before initiating the surgery, all five screw electrodes and the ground one are soldered to golden plated sockets (black arrow) via an insulated copper wire. The wires are lightweight and moldable in various shapes which facilitates handling during surgery and giving the headset its final shape. **B** Shown is the 6-channel plastic pedestal (white arrow) that accommodates the 6 electrode sockets. After placing the sockets in their respective ports, the pedestal is pushed down while tucking in the copper wires in order to form a compact headset. **C** Dental cement is poured from the sides using a syringe, while still in a semi-liquid form in order to allow infiltration of all the gaps between the wires and pedestals. This gives the protective headset its final form that keeps all the elements together, and protects the skull, without requiring sutures. **D** After 5 days of postoperative resting, the headset is connected to the EEG cables (white arrow) ending with 6 pins that fit into the pedestal on the headset. The 6 pin EEG cable, a pedestal, and a screw electrode connected to a socket, are shown next to each other for illustrative reasons
5. The rats are placed on a warming pad (36–37 °C) during the entire surgery. After the surgery, they are placed in the special customized single EEG cages for post-operative observation. Analgesia regimen with paracetamol (1 mg/ml in drinking water) is administered for 3 days postoperatively [18]. Rats are monitored daily after the surgery and during subsequent EEG recordings by experienced trained personnel. At any sign of infection including discharges around the EEG head cap or the surgical site, decreased feeding or mobility, rats are humanely euthanized using the anesthetic mixture described in step 1 above, followed by placement in the CO_2_ chamber.
6. After a 5-day recovery period, the EEG cables ending with a 6-pin plug (Fig. 1D) are attached to the 6-channel pedestal, and EEG recordings are initiated.

Various rodent seizure models are utilized in our translational epilepsy laboratory depending on the research paradigm. Seizures in the kainic acid model of temporal lobe epilepsy and in the hypoxic seizure model are induced as previously described [10, 19]. For the closed head TBI models, we adapted the methods published by Yang et al. [20]. Following EEG recordings, rats receive the same intramuscular mixture of anesthetic described in step 1 above, and brains are perfused with paraformaldehyde via the cardiac route in order to perform various histological studies.

### Customized EEG cages and the swivel-balance system

#### Cages and racks

**Fig. 2:**
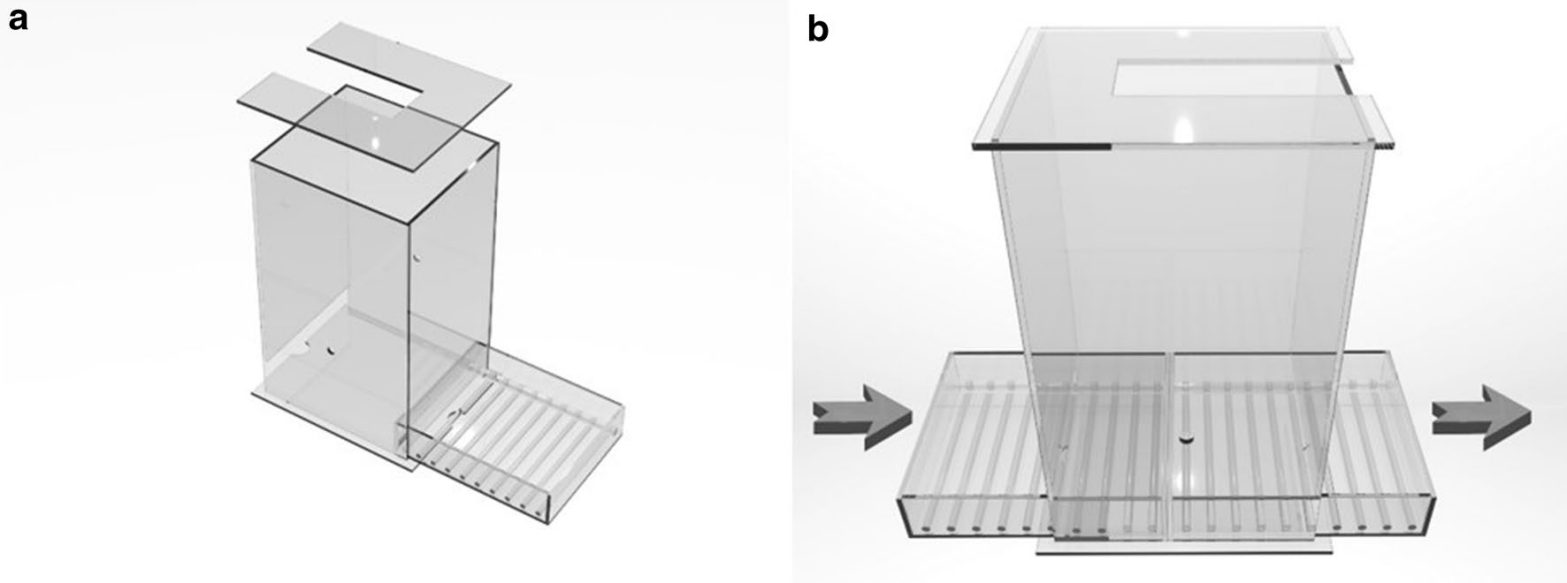
Customized plexiglass cage. **a** Shown is the customized plexiglass cage and its various parts, including the gridded floor and the removable ceiling. The gridded floor consists of a drawer that contains the bedding and a side container for food. The gridded floor helps maintaining an even distribution of bedding over the floor. **b** Shown is a schematic representation of the sliding drawer. The food and bedding are replaced by sliding in a clean drawer as the old one is pushed out. This simple system allows bedding and food changes without disconnecting the rat or removing it from the cage

**Fig. 3:**
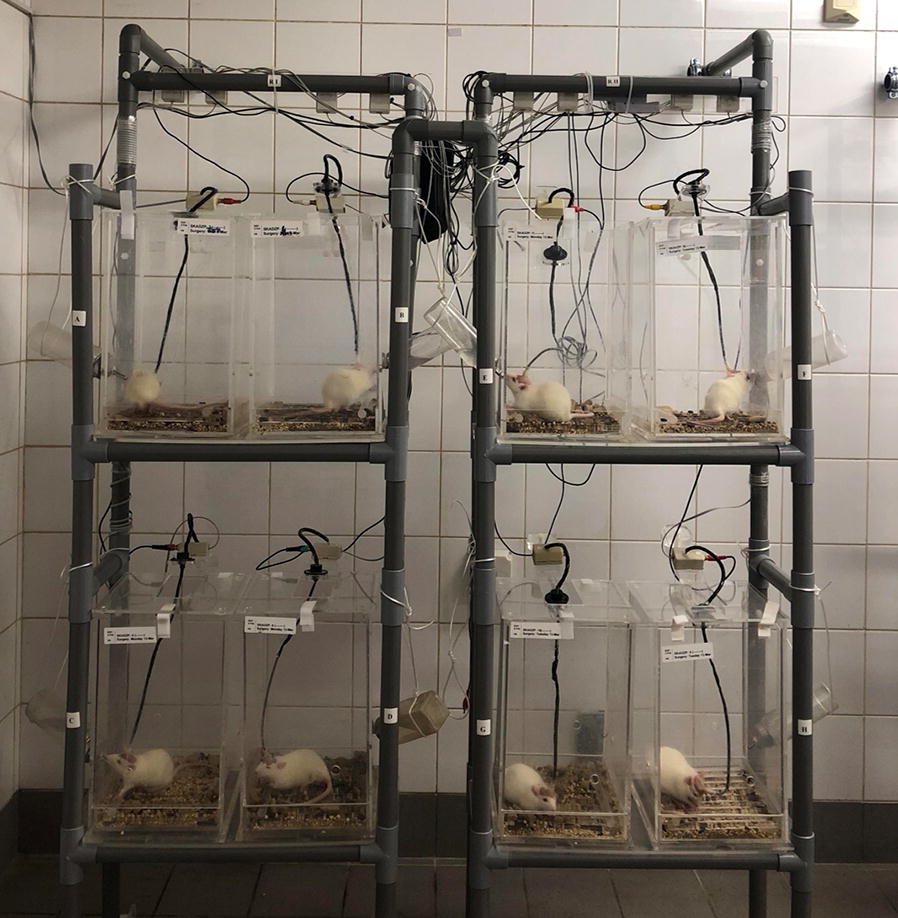
EEG rack system. Each rack houses 4 cages, two on each shelf. Every 2 racks (8 cages) are monitored by one EEG recorder. We built a rack system that does not contain metal or other conductive material which provides the simplest and most efficient technique to electrically isolate rats from each other and from environmental sources of electricity. This has markedly contributed to minimizing electrical artifact, which is a challenge commonly faced when simultaneously recording multiple animals

Rats are individually placed in customized plexiglass EEG recording cages (22L × 22W × 36H cm). These are equipped with sliding drawer-like gridded floors in which wood shaving bedding is easily changed by sliding a clean drawer in, while pushing the used one out (Fig. 2). The drawer allows replacing the dirty bedding and providing food without removing the animal from the cage (Fig. 2b). The grid helps in maintaining the wood shavings homogeneously distributed over the floor of the drawer. Food is placed on the side of the gridded floor in a special container. The ceiling of the cage consists of a removable plexiglass lid which has a rectangular opening (Fig. 2). This permits the vertical movement of a pivoting rectangular plate that holds a swivel above the cage and a weighted counterbalance on the other side as described below. Similarly to the cages, the rack system that houses them, is also built from electrically nonconductive material by assembling plastic pipes and connectors, providing electrical isolation for individual rats from each other and from sources of environmental electricity. Each rack has 2 plexiglass shelves and is designed to accommodate 4 cages (Fig. 3). Water bottles are held in place with plastic wires fastened to the rack, and are accessed by the rats via holes drilled on the sidewall of the cages.

#### Swivel balance and cable system

**Fig. 4:**
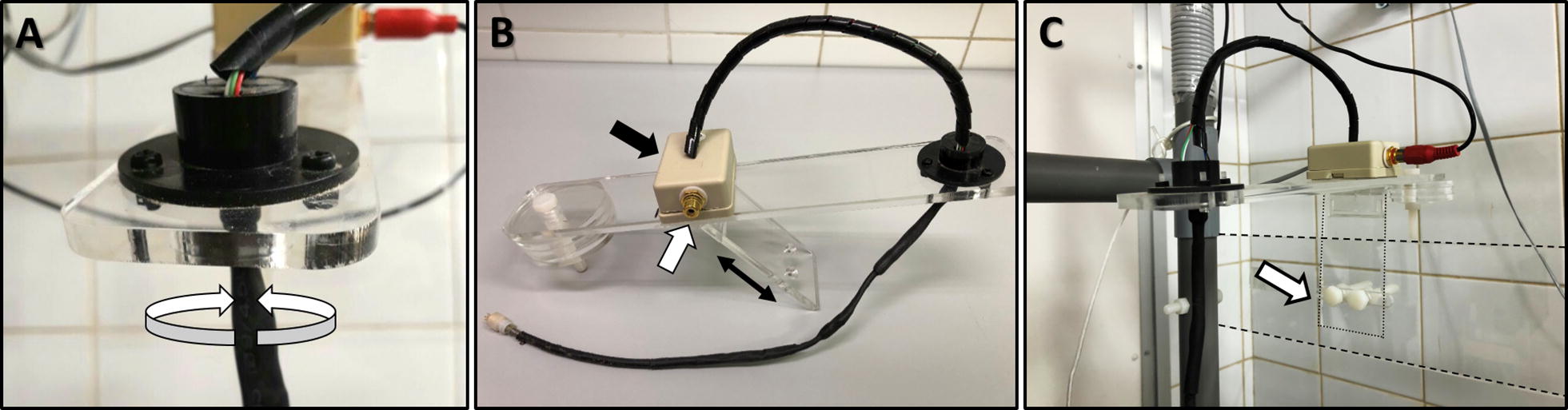
Elements of the swivel balance arms. **A** The standard swivel consists of a center rotating spindle and six conductor rings in electrical contact via conductor brushes. The used swivel accommodates the EEG cable carrying electrical signal from the 6 electrodes implanted in each rat. It has a low torque turning resistance preventing torsion of the EEG cable and subsequent stress on the rat’s headset. This standard swivel prevents cable disconnection by allowing torsion-free motion of the rat in the horizontal plane. The curved white arrows indicate the rotating mobile part. **B** The swivel is fixed on a balance that consists of a plexiglass rectangular plate (30 cm × 5 cm) that pivots along a hinge plexiglass plate (10 cm × 5 cm, double arrows). The EEG cable ending with the 6-pin plug goes through the swivel and ends up in a relay box that separates the reference and ground signals (RCA plug, white arrow) from those of the 4 sampling electrodes. **C** The hinge plate is attached to the back of the plexiglass cage rack (area between the 2 dashed lines) with 2 plastic screws (white arrow). The relay box is attached to an RCA cable (carries signal from ground and reference electrodes) and to an ethernet cable on the opposite side (carries electrical signal from the 4 sampling electrodes)

**Fig. 5:**
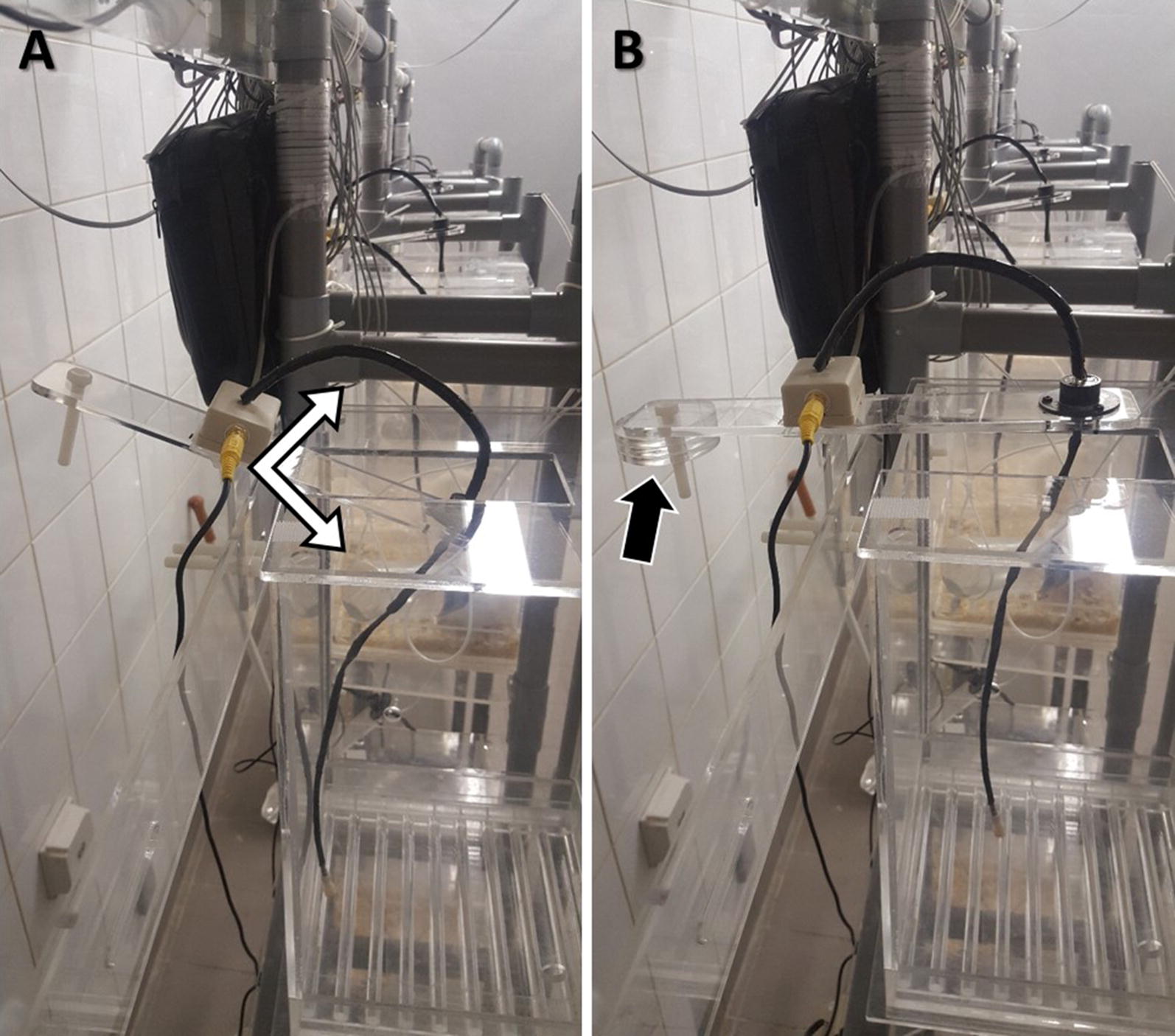
Swivel balance cage system. We have built a simple system that prevents tension and torsion on the EEG cable as the rat freely moves in both the horizontal and vertical planes. The mobile rectangular plate that holds the standard swivel can move up and down through the cage’s ceiling as it pivots along a hinge (**A** double arrows). The cage is placed on the shelves so that the swivel is centered over it. The swivel is counterbalanced by circular weights (**B** black arrow) placed at the other end of the plate. The circular weights are loosely screwed via non-concentric holes allowing easy balancing by just turning the weights around which alters the torque they exert on the plate to the desired effect. Prior to attaching the animal to the cable, the weights are calibrated so that the counterbalance arm holding the weights is slightly heavier (**B**). This will provide a minimal continuous upward pull on the headset when the animal is attached, and prevent the swivel from remaining inside the cage when no downward tension is exerted by the animal, as occurs when the rat rises. These simple yet key modifications allow long-term continuous EEG monitoring without premature disconnections of the EEG cable from the rat’s head

We have built a cage and wiring system with a special attention to the most fragile parts; the components connecting the headset to the swivel, especially those in contact with the rat’s head. A standard swivel (torque: 0.06 Nm) allows rotation of the EEG cable as the rat moves in the horizontal plane. In addition to allowing the torsion-free animal motion in the horizontal plane provided by the standard swivel, our customized swivel-balance system also allows free movement in the vertical plane. Given that the rat’s head movements are also vertical exerting pulling tension on the EEG cable, we built a swivel system that can move up and down to prevent tension when the rat is in the resting position, and also preventing cable twisting inside the cage when the rat moves up (Fig. 4). This was achieved by fixing the swivel on a rectangular plate that pivots vertically along a hinge neck placed on the back wall of the rack allowing the swivel to move up and down through the rectangular opening in the ceiling (Fig. 5A). The plate pivots along the hinge placed in its middle with one arm holding the swivel above the center of the cage on one side, and an external arm holding counterbalancing weights on the other side. These weights are placed on the external arm of the plate so that the weights of the cable and swivel of the inner arm are counterbalanced, with a net 0.02 N upward vertical force on the swivel. This gentle upward force on the swivel prevents it from staying inside the cage (with subsequent EEG cable folding) when the rat is not pulling down on it (Fig. 5B). In addition, the EEG cables connecting the headset to the swivel inside the cage are also protected against chewing with stainless steel spring covers. The length of the EEG cable hanging inside the cage is 32 cm (swivel to rat’s headset). This length was calculated based on the cage dimensions in order to prevent cable tension when the rat is in the corner, and at the same time to avoid excessive cable folding, and thus increased exposure to chewing, when the rat is in the center of the cage.

### Digital EEG recording system

**Fig. 6:**
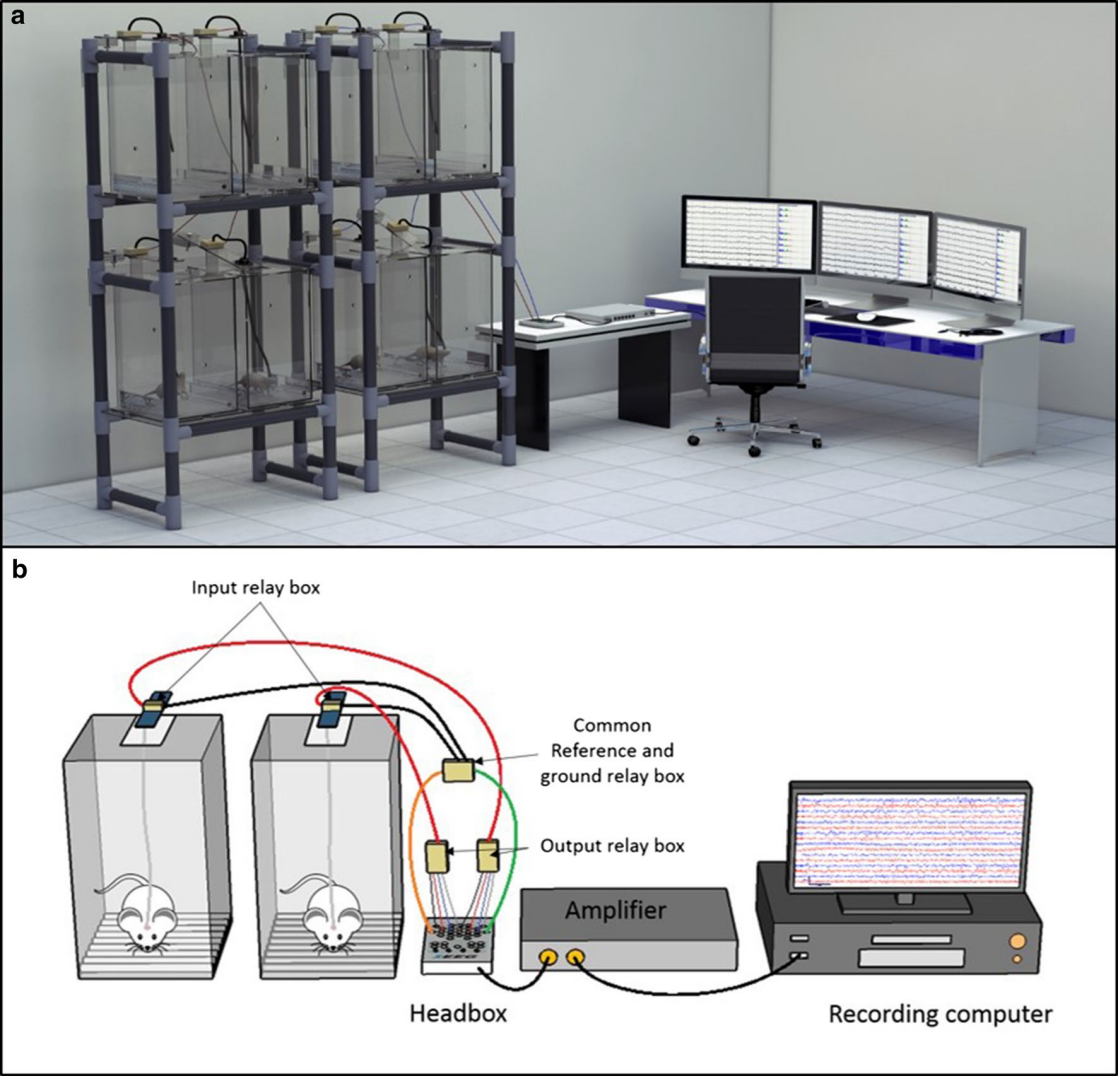
A representation of the entire recording system. **a** Schematic illustration of the full two rack system. **b** The EEG cable coming through the swivel is connected to an output relay box placed in the middle of the pivoting rectangular plate, centered over the hinge. The output relay box simplifies the wiring by splitting the signal into two cables only, the red cable carrying the epidural cortical or depth electrode signal (4 per rat), and a black cable carrying signal from the ground and reference electrodes. The red and black cables are then connected respectively to the input relay box and to the common reference/ground relay box. The input relay box splits again the electrical signal of each rat into its 4 epidural cortical or depth electrode wires, and these are connected to 4 ports on the headbox. Given that a single reference port and a single ground port are available on the 32 channel digital headbox (Xltek, Natus Medical, USA), the common reference/ground relay box combines reference and ground electrode signal from 8 rats into one common reference and one common ground respectively, which are placed in their respective headbox ports. The headbox feeds data into an amplifier that is connected to the recording computer. The used cable system and relay boxes minimize the number of wires for easy access and troubleshooting if needed. EEG tracings can be acquired simultaneously on 8 rats using one computer recorder and one headbox. Only two cages are shown for illustrative simplicity

In order to simplify the setup, wires connecting the swivel to the EEG recording headbox (32 channel headbox, XLTEK, Natus Medical, USA) go through output and input relay boxes as described in details in Fig. 6. Relay boxes are used in order to minimize the number of wires which not only improves organization but also the ability to readily access cables and to systematically troubleshoot the various components of the system when needed. The four epidural cortical electrodes or depth electrodes originating from one rat’s headset are ultimately connected to four different ports on the 32 channel headbox which can accommodate up to 8 rats. The reference electrodes of the 8 animals are daisy chained and connected to the only available reference port in the headbox. The 8 ground electrodes are also approached in the same manner and connected to the single ground port on the headbox. The headbox feeds data into an amplifier (Connex amplifier, XLTEK, Natus Medical, USA, 512 Hz sampling frequency, and 16 bits sampling resolution) providing monopolar referential EEG recordings. The amplifier is connected to a recording computer, and allows the simultaneous continuous recording from 8 animals using one headbox. The grounding and referencing approaches are designed to maximize the signal to noise ratio while overcoming the limitations of the standard medical headbox that allows input from only one reference electrode and only one ground electrode as it is designed for a single human subject. In addition to the usually clinically used bipolar transverse and bipolar longitudinal montages, the Laplacian montage option is used to create an average referential montage for each rat (comparing the input of each of the four epidural or depth electrodes from one animal to the average signal of these four). Chronic continuous EEG tracings are briefly discontinued and restarted on the recording computer every 48–72 h in order to minimize EEG file sizes and facilitate the organization of the sizeable EEG data review process.

## Results

Using the herein described long-term EEG monitoring unit setup, we were able to perform chronic EEG simultaneously on up to 16 rats (8 rats per EEG recorder) in order to successfully complete various experimental paradigms in different animal models of seizures. We have so far recorded more than 150 rats between the ages of P35 and P150 belonging to various experimental models (Table 1) for up to 3 months, without suffering from interruptions or EEG cable disconnections (Additional file 1). Of interest, irrespective of the length of the experimental paradigms, there were no unplanned recording interruptions or premature terminations due to cable detachments or any other mechanistic technical issues. Furthermore, we have not encountered any scalp or bodily injuries in rats undergoing recordings demonstrating utility and applicability of this system in juvenile and adult rats. The only 3 unplanned EEG cable detachments were due to cable chewing by relatively large older rats, more than 2 months through the prolonged EEG recording, at around 140 days of age. This has prompted us to cover the whole EEG cable hanging inside the cage with a protective metal spring cover (as opposed to only the lower third of the cable in the initial setup). The counterbalancing weights of the external arm of the swivel-balance cage system were adjusted to account for the new weight (10 g) of the EEG cable with its extended cover spring. In addition to the mechanical advantages of accommodating movements conferred by the swivel-balance cage system, tightly securing screw electrode to the calvarium also contributes to the prevention of premature EEG headset detachments. As shown in Table 1, our setup accommodates even the notoriously vigorous kainic-acid induced seizures that consist of repetitive rhythmic motoric activity (Racine stage 3–5) often continuously or in a recurrent manner over up to 2–3 h (status epilepticus) in the initial acute seizure induction phase.

**Table 1:** Successful applications of the swivel-balance EEG cage system

Age at time of recording initiation	Number of animals	Acquired seizure model	Duration of long-term EEG recording	Reason for EEG termination	Premature disconnections
P45–55	40	Hypoxic seizures	3 months	Experimental protocol	Two unplanned premature disconnections due to cable chewing
P45	2	Kainic acid model of TLE	3 months	Experimental protocol	None
P45–50	20	TBI	1–2 months	Experimental protocol	None
P45	120	Kainic acid model of TLE	24–72 h	Experimental protocol	None

Shown in this table are illustrations of various successful applications using our EEG setup in animal models of acquired seizures. Recordings are started in male Sprague–Dawley rats at 40–45 days of age (P40–45) with a weight range of 200–250 g. The recordings are performed on multiple rats simultaneously (8 rats per EEG recorder) for up to 2–3 months (weights reaching up 500 g) as dictated by the set experimental paradigms. So far, there were only 2 premature disconnections around P140 due to chewing of the cable by rather older rats. This problem was remediated in subsequent recordings with placing metal spring covers on the EEG cable hanging inside the cage (*TLE* temporal lobe epilepsy)

**Fig. 7:**
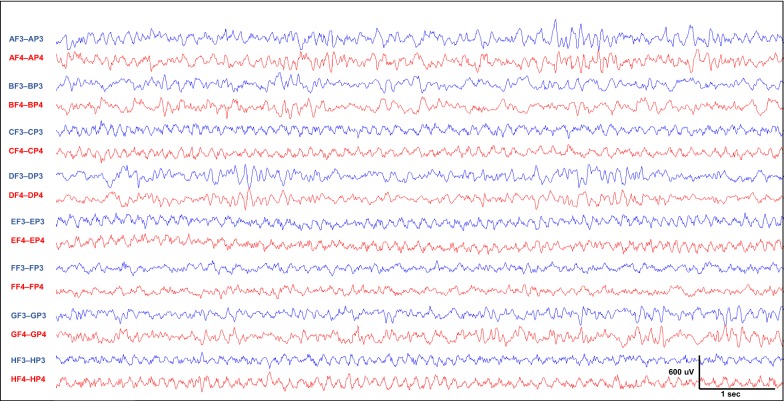
Simultaneous monitoring of 8 rats with continuous long-term EEG. Shown is a screenshot of a continuous EEG recording performed simultaneously on 8 P100 rats using one EEG recorder. This screenshot was obtained 2 months after starting a long-term recording on rats that underwent early life hypoxic seizures, and reveals the high quality and stability of the recording system and wiring over prolonged periods of time. Each alphabetical letter refers to a rat subject. Shown is a longitudinal bipolar montage with 2 channels per rat (each channel represents the voltage difference between the frontal and parietal sampling electrodes, F3: left frontal, P3: left parietal, F4: right frontal, P4: right parietal). This EEG reveals a normal tracing for all the rats. Tracing of rats C, E, and H are consistent with the 6 Hz theta rhythms seen in relaxed rats, likely a thalamocortical feature that recapitulates the human posterior dominant rhythm given the rhythmicity and monomorphism. The EEG tracings of rats A, B, D, F and G shows drowsy and sleep rhythms. The notch was set at 50 Hz

**Fig. 8:**
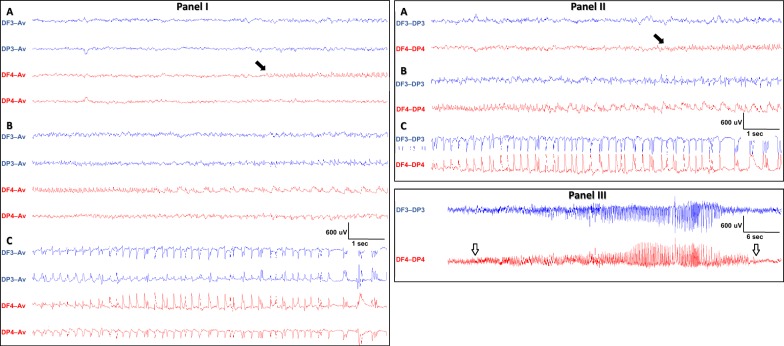
Seizure onset localization using an assortment of montages. Illustrative successive screenshots (A, B then C) of an evolving right frontal seizure in an average referential montage (Panel I) and in a longitudinal bipolar montage (Panel II). This is a typical seizure with “a rhythmicity that evolves in space and time”. It starts in the right frontal area (black arrow) as shown in screenshots A, then progresses to involve the whole right hemisphere with relatively slower rhythmic activity (screenshots B), and finally clearly involves the left hemisphere with bilateral rhythmic spikes (screenshots C). In Panel III, the entire almost 40 s seizure is shown on a larger timebase (white arrows indicate seizure onset and offset). This high quality and artifact-free recording was stable and not affected by the seizure even though it was accompanied by full body generalized tonic–clonic movements (Racine stage 5). In this P40 rat, with kainate-induced status epilepticus, seizures similar to the one shown here, were near continuous over a period of 4–6 h, and then frequently recurred in the first 24 h following the kainic acid administration in this model of temporal lobe epilepsy. The vigorous movements associated with kainate-induced status epilepticus did not affect the electrical continuity, stability, or quality of the EEG tracings. F3: left frontal, F4: right frontal, P3: left parietal, P4: right parietal, Av: average of the signals obtained from the 4 sampling electrodes

Our EEG setup allows us to monitor EEG recordings of 8 rats simultaneously using one EEG recorder and one monitor showing an 8 rat display (Fig. 7), and this allows the time-efficient review of sizeable data spanning months of EEG recordings. As shown in Fig. 7, the recordings are of a high quality and artifact-free despite multiple simultaneous prolonged tracings acquired for up to 3 months. In addition to the mechanical advantage of maintaining electrical continuity described above, the sustained high signal-to-noise ratio over long periods of recordings is also the result of appropriate electrode grounding and referencing, as well as efficient electrical shielding of rats from each other and from ambient electrical noise with nonconductive plexiglas cages housed on plastic racks. In the absence of prominent movement or ambient electricity-related electrical interferences, epileptiform activity can be easily detected and even localized to a specific brain area in these high quality tracings. Recurrent and even motorically vigorous seizures did not affect the quality of the tracings and their ictal onset were also easily localized to specific brain areas (Fig. 8).

## Discussion

Here we describe in details an enhanced EEG monitoring unit setup to record continuous EEG simultaneously on multiple animals for up to 3 months without putting the rats at risk of injuries and without EEG cable disconnections and premature experimental terminations. The improvements applied to the wired cage system and the attention to details of electrical shielding, electrode grounding and referencing, allowed us to perform long-term EEG recording without resorting to wireless radio-telemetry; a technology that remains very expensive at this point in time. We believe that the special attention to the most vulnerable parts of the EEG recording system, namely the components between the rat’s head and the swivel was key in enhancing the recording system. Indeed, the vertically mobile swivel-balance system prevents torsion due to motion in the horizontal plane as well as tension imparted by vertical movements of rats. The delicate balancing of the counterweights was also vital in providing a continuous gentle upward pull on the rat’s headset, preventing the folding of the EEG cable inside the cage which exposes it to the natural tendency of rats to chew on it. In addition, tightly securing the screw electrodes to the calvarium is essential, since the screws serve as the main anchors that attach the EEG cable to the rat’s head. On the other hand, the dental cement is merely the “glue” that keeps together the electrodes, wires, and other components of the headset and shield them from the external environment. Furthermore, the use of a sliding drawer approach to change the bedding and place food while the rat remained connected to the EEG cable inside the cage, minimized frequent EEG cable connections and disconnections, and attenuated the mechanical wear and tear of the cables and headset, offering a sustained integrity of electrical continuity. These modifications are simple-to-implement, and offer major quality and longevity advantages over the current wired EEG cage system.

In addition to preventing premature cable disconnections by markedly attenuating the above described various sources of mechanical disruptions to electrical continuity, the attention to electrical shielding of rats, and to electrode wiring and grounding has resulted in high quality artifact-free EEG tracings (improved signal-to-noise-ratio). The use of nonconductive material in almost all the elements of the long-term EEG rats’ unit, including the plexiglass cages, plastic rack system, high pressure laminate (HPL) trays and HPL computer recorder tables, has facilitated the grounding process and the electrical shielding of the rats from each other and from ambient sources of electrical noise. Grounding and referencing in this electrically shielded unit was performed without difficulties via a daisy chain approach, overcoming the commonly challenging task of simultaneously recording multiple animals using medical devices tailored to one human subject.

## Conclusions

The herein described long-term EEG rat monitoring unit provides a simple and cost-effective approach to acquire continuous high-quality EEG tracings simultaneously on multiple rats for several months without disconnections or injuries. The customized cages, housing environment, and the swivel-balance system can be easily constructed using relatively inexpensive materials and tools. The described detailed methodological approach to electrode implantation and wiring can also be easily implemented. One of the limitations of our setup, is the lack of electromyography (EMG) that can provide additional information to video-EEG especially in the case of behaviorally subtle seizures. Muscle leads can be added to this system by using the differential input on the headbox allowing muscle recordings from up to 6 rats (one channel per rat). In addition, our system still has the limitation imposed by tethering, and thus, unlike wireless techniques [21], cannot record from freely moving rats undergoing behavioral testing or from pups caged with their mother (younger than 21 postnatal days). Nevertheless, our simple-to-implement key modifications result in a markedly improved traditional hardwired EEG and provide the necessary quality and amount of data required for the long-term study of seizure chronobiology in juvenile and adult rats [22] at a much lower cost than more advanced wireless systems.

## Additional file

**Additional file 1.** Illustrative video of a continuous EEG recording in a rat using the swivel balance cage system. The system accommodates all sorts of rat’s movements to the cage’s corners, as well as climbing, jumping, turning around, reaching for food, and grooming.

## Abbreviations

EEG: electroencephalogram
HPL: high pressure laminate
P: postnatal day
PTE: post-traumatic epilepsy
TBI: traumatic brain injury
TLE: temporal lobe epilepsy
